# Diagnosing Dysphagia in Forestier Syndrome: A Dynamic Digital Radiology Application

**DOI:** 10.3390/diagnostics15233020

**Published:** 2025-11-27

**Authors:** Michaela Cellina, Daniele Bongetta, Carlo Martinenghi, Giancarlo Oliva

**Affiliations:** 1 Radiology Department, ASST FBF SACCO, Fatebenefratelli Hospital, Piazza Principessa Clotilde 3, 20121 Milan, Italy; 2 Neurosurgery Department, ASST FBF SACCO, Fatebenefratelli Hospital, Piazza Principessa Clotilde 3, 20121 Milan, Italy; 3 Radiology Department, San Raffaele Hospital, Via Olgettina 60, 20132 Milan, Italy

**Keywords:** DDR, Forestier syndrome, dysphagia, dynamic imaging, swallowing study

## Abstract

Diffuse idiopathic skeletal hyperostosis (DISH), or Forestier’s disease, is a non-inflammatory condition characterized by the calcification and ossification of spinal ligaments and entheses, especially the anterior longitudinal ligament. Its prevalence increases with age and it is more common in males. The term DISH usually refers to the imaging aspects of this condition, while “Forestier’s disease” is used for the clinical correlates of the condition, especially the development of dysphagia. Diagnosis is usually made with conventional radiography, based on the Resnick and Niwayama criteria: flowing osteophytes over at least four contiguous vertebral bodies, the preservation of intervertebral disk space, absent facet and costovertebral joint ankylosis, and absent sacroiliac joint abnormalities. A “melted candle wax” appearance along the spine is typical of the advanced disease. Large anterior osteophytes in the cervical spine lead not only to stiffness and chronic neck pain, but also to compressive symptoms such as dysphagia, dysphonia, and even airway compromise. Digital Dynamic Radiography (DDR), thanks to a flat-panel detector system, captures high-temporal resolution sequential low-dose radiographs at high frame rates in dynamic motion studies to provide functional information. We report the case of a 50-year-old female patient diagnosed with Forestier’s disease. Cervical radiography showed coarse anterior osteophytes and calcifications typical of DISH. The patient complained about persistent cervical pain and significant dysphagia. To investigate the underlying mechanism, a DDR with barium oral administration was performed. The examination confirmed the mechanical narrowing of the pharyngeal lumen caused by bulky anterior osteophytes. Given the severity of the symptoms, the patient underwent a surgical resection of the osteophytic and calcified components, with a subsequent improvement of swallowing function. This case highlights how DDR provides functional and morphological information in patients with dysphagia related to cervical DISH.

**Figure 1:**
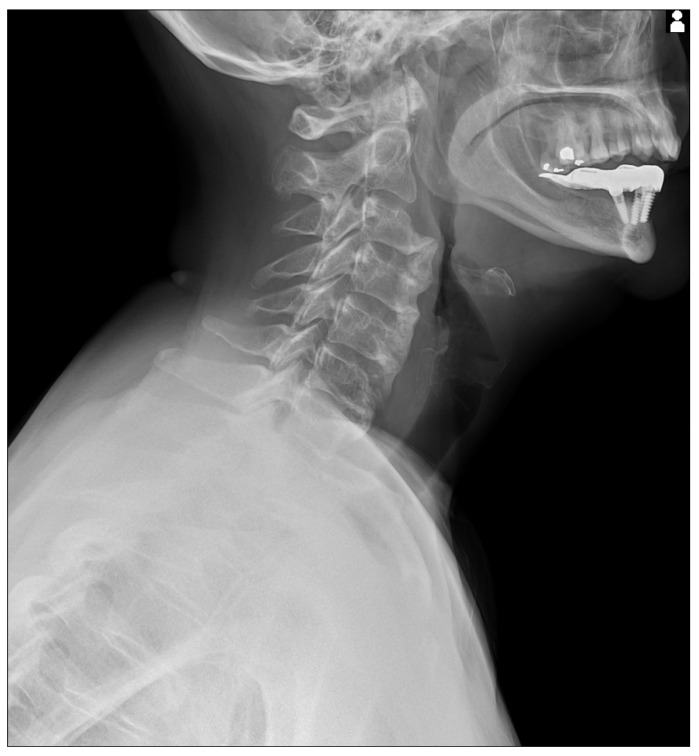
Static X-Ray acquisition acquired with Konica Minolta (AeroDR TX, Konica Minolta Inc., Tokyo, Japan) with a static setting. The cervical X-Ray shows florid, flowing ossification along the anterior or right aspects of five contiguous vertebrae, from C3 to C7 [1,2,3,4,5,6]. The intervertebral disk spaces are preserved.

**Figure 2:**
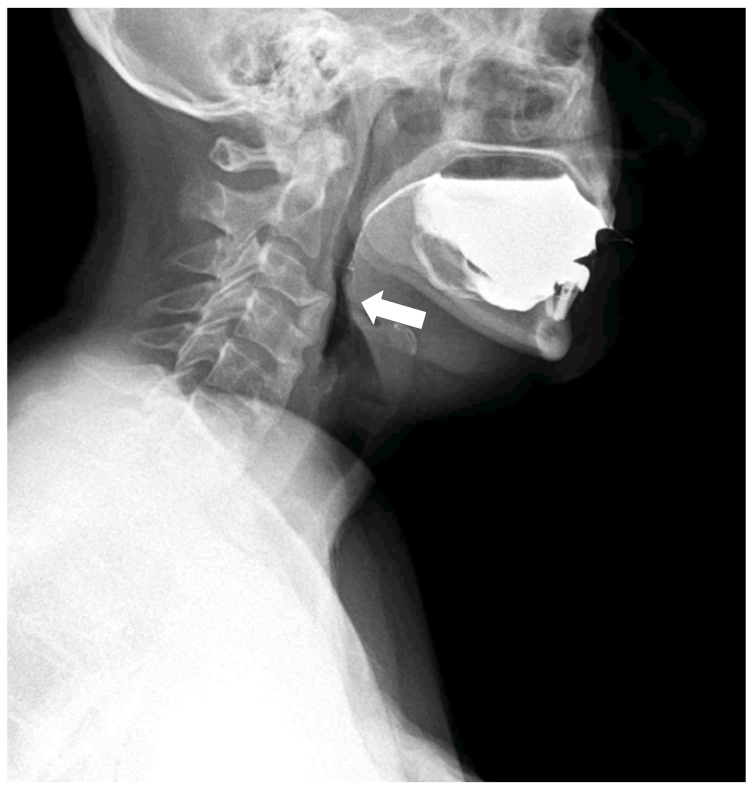
Dynamic swallowing study (Konica Minolta, AeroDR TX, Konica Minolta Inc., Tokyo, Japan) during the administration of 30 mL barium and water in the laterolateral view. The distance between the tube and detector was set at 120 cm. Acquisition parameters: tube voltage, 60 kV; tube current, 80 mA; 15 fps, according to a previous study [7]; with acquisition time of 28 sec. The huge osteophyte of C3, associated with the coarse calcification of the anterior ligament, results in a narrowing of the pharyngeal lumen (arrow).

**Figure 3:**
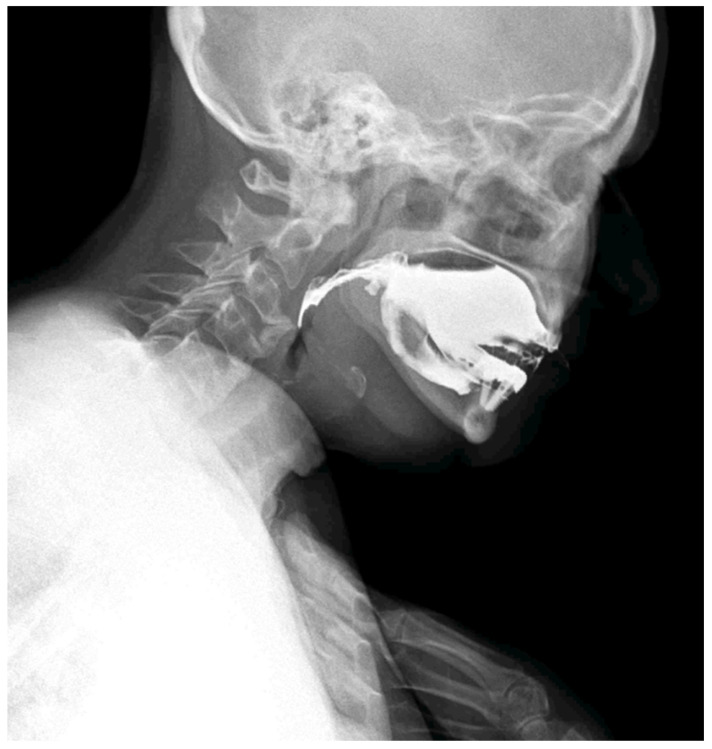
The patient adopted a compensatory swallowing position with neck and chest flexion forward to reduce pharyngeal narrowing and facilitate bolus passage. The pharyngeal lumen is narrowed by the anterior cervical osteophytes. Compared with traditional videofluoroscopic examinations, DDR offers a wider field of view, quantitative motion analysis, and significantly reduced radiation exposure [8,9,10]. In the context of swallowing disorders, DDR allows simultaneous evaluation of bolus transit and the impact of mechanical obstructions, while also detecting compensatory strategies adopted by the patient. Moreover, DDR technology is available on portable equipment; this allows the clinicians to conduct a swallowing study directly at the patients’ bed, which is particularly important when dealing with frail patients affected by dysphagia and silent aspiration. The whole dynamic acquisition captured at 15 frames/s is presented in Supplementary Material S1.

**Figure 4:**
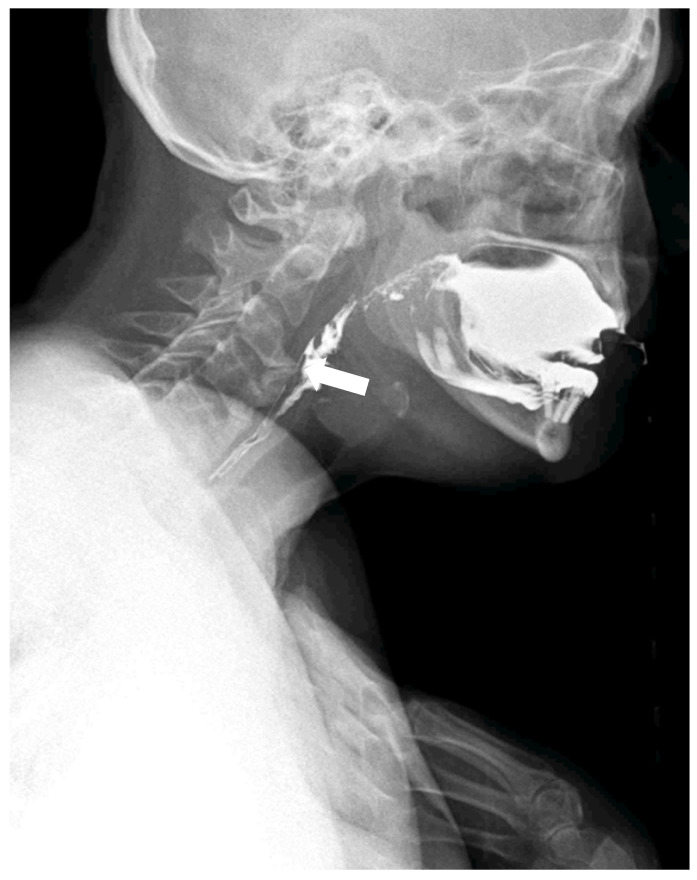
The compensatory position with the anterior flexion of the neck and chest is effective in slightly reducing the compressive effect of the osteophyte and ligament calcification to enable the contrast bolus passage (white arrow).

**Figure 5:**
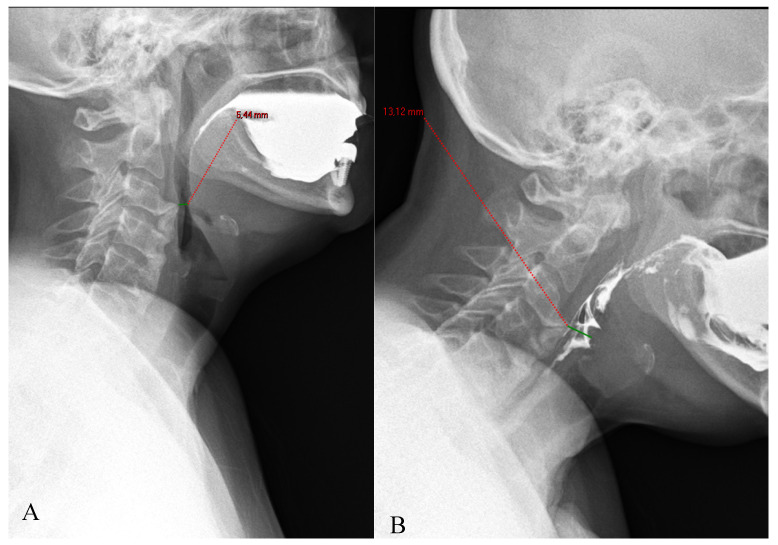
The compensatory position with anterior neck flexion (**B**), allows the patient to increase the pharyngeal diameter at the level of the narrowing related to the C2 osteophytes, compared to the neutral neck position (**A**).

**Figure 6:**
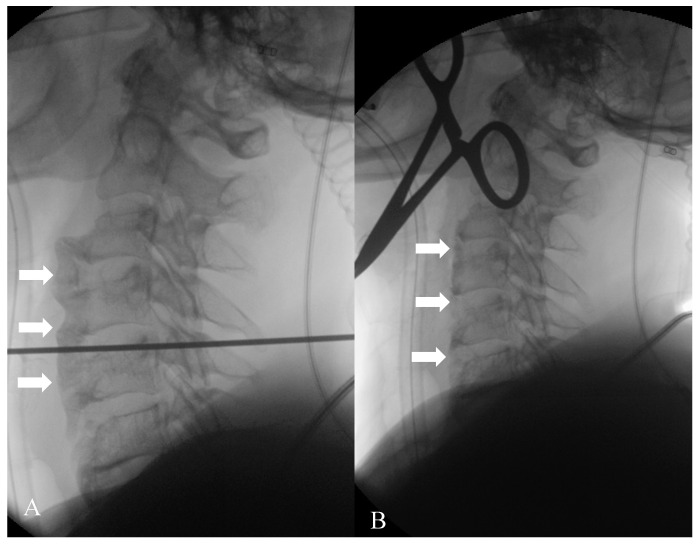
Given the severity of the symptoms, the patient underwent a surgical resection of the anterior ligament calcification and osteophytes. (**A**) represents the beginning of the surgical procedure, with evidence of multiple osteophytes and anterior ligament calcifications (arrows); (**B**) is the image acquired at the end of the intervention, where they are no longer recognizable (arrows).

## Data Availability

This is just the description of a case, without any additional available data.

## Supplementary Materials

The following are available online at https://www.mdpi.com/article/10.3390/diagnostics15233020/s1, Video S1: Dynamic swallowing study. The patient spontaneously adopted a compensatory swallowing strategy with neck flexion, which partially reduced pharyngeal narrowing and facilitated bolus passage. The pharyngeal lumen is narrowed by the anterior cervical osteophytes.

## Abbreviations

The following abbreviations are used in this manuscript:

DISH	Diffuse idiopathic skeletal hyperostosis
DDR	Dynamic Digital Radiography
